# Non-invasive monitoring of microcirculation dynamics in hypovolemic shock: a novel application of diffuse correlation spectroscopy

**DOI:** 10.1186/s40635-025-00761-9

**Published:** 2025-06-01

**Authors:** Hiroki Matsushita, Koki Kurono, Mikie Nakabayashi, Kei Sato, Hidetaka Morita, Yuki Yoshida, Masafumi Fukumitsu, Kazunori Uemura, Toru Kawada, Masashi Ichinose, Yumie Ono, Keita Saku

**Affiliations:** 1 Department of Cardiovascular Dynamics, National Cerebral and Cardiovascular Center Research Institute, 6-1 Kishibe-Shimmachi, Suita, Osaka Japan; 2 Graduate School of Science and Technology, Meiji University, Kawasaki, Kanagawa Japan; 3 NTTR-NCVC Bio Digital Twin Center, National Cerebral and Cardiovascular Center, Suita, Osaka Japan; 4 Human Integrative Physiology Laboratory, School of Business Administration, Meiji University, Tokyo, Japan; 5 School of Science and Technology, Meiji University, Kawasaki, Kanagawa Japan

**Keywords:** Microcirculation, Hypovolemic shock, Diffuse correlation spectroscopy, Blood flow index

## Abstract

**Background:**

Microcirculatory dysfunction is a poor prognostic indicator for the management of critically ill patients, highlighting the need for the development of appropriate assessment methods. Current microcirculatory parameters are often indirect, invasive, or lack immediacy and continuity, with no standardised markers for critical care. Diffuse correlation spectroscopy (DCS), a near-infrared optical technique, facilitates the non-invasive real-time monitoring of microvascular dynamics via the blood flow index (BFI). However, the relationship between BFI and conventional microcirculatory parameters in hypovolemic shock remains unclear. This study examined the utility of DCS in assessing the microcirculation during hypovolemic shock in a canine model.

**Methods:**

Six male beagle dogs underwent controlled blood withdrawal to induce hypovolemic shock, defined as a ≥ 30% decrease in cardiac output (CO) and mean arterial pressure (MAP) < 60 mmHg or systolic arterial pressure (SAP) < 90 mmHg. BFI was measured using a DCS device attached to the skin of the forelimb. From baseline to blood withdrawals followed by transfusions, changes in BFI were compared with microcirculatory parameters, mixed venous oxygen saturation (S_v_O₂), core-to-skin temperature gradient (ΔT), veno-arterial difference in partial pressure of carbon dioxide (PCO₂ gap), and serum lactate. Correlation and receiver operating characteristic (ROC) analyses were performed to determine the cut-off value of relative BFI for predicting whether lactate levels exceeded 22.5 mg/dL.

**Results:**

Blood withdrawal resulted in significant reductions in BFI, CO, and radial artery blood flow, with the corresponding deteriorations in the ΔT, S_v_O₂, and PCO_2_ gap and lactate levels. BFI showed significant correlations with ΔT (correlation coefficient [CC] = − 0.48, 95% confidence interval [CI] − 0.69 to − 0.18, *p* < 0.01), SvO₂ (CC = 0.67, 95% CI 0.43 to 0.81, *p* < 0.01), and PCO2 gap (CC = − 0.63, 95% CI − 0.79 to − 0.39, *p* < 0.01). ROC analysis identified a relative BFI threshold of 35.5% of the baseline for predicting elevated lactate levels, with 62% sensitivity and 100% specificity (AUC = 0.75).

**Conclusions:**

Blood flow index measured by DCS reflects peripheral perfusion changes and is significantly correlated with clinical parameters during blood withdrawal and transfusion, highlighting its potential for non-invasive, continuous microcirculation monitoring in hypovolemic shock.

## Background

Haemorrhage triggers sympathetic activation, causing peripheral vasoconstriction and increased ventricular contractility, which redistribute blood flow. This maintains perfusion to vital organs during the compensatory phase of shock, while reducing flow to peripheral tissues like the skin and muscles [1]. Reduced perfusion promotes platelet activation and contributes to capillary obstruction through endothelial and neutrophil activation [2]. Persistent microcirculatory impairment can lead to critical oxygen deficiency and irreversible shock [3, 4]. Recent studies show that macrocirculatory and microcirculatory functions can become uncoupled during shock [5]. In hypovolemic shock, microcirculatory dysfunction occurs early and may persist despite normalised systemic parameters [6, 7] and has been linked to multiple organ failure in trauma patients [8]. Early microcirculatory monitoring, alongside conventional parameters, may improve assessment and guide treatment. Thus, establishing reliable microcirculatory indices in hypovolemic shock is both essential and urgent.

Microcirculation is typically assessed through capillary refill time, urine output, the core-to-skin temperature gradient (ΔT), serum lactate, and mixed venous oxygen saturation (S_v_O₂) [9]. These parameters have significant limitations, including invasiveness, delayed results, lack of continuity, and an indirect reflection of tissue metabolism rather than real-time blood flow. Although direct methods, such as handheld vital microscopy, provide visual insights into pathological microcirculatory changes [10], the complexity of their analysis has hindered their widespread clinical use. Therefore, standardised or reliable microcirculatory parameters have yet to be established, highlighting the urgent need for non-invasive quantitative tools [11].

Diffuse correlation spectroscopy (DCS) has emerged as a promising optical technology, using near-infrared light to non-invasively monitor microvascular blood flow in deep tissues [12]. DCS quantifies microvascular blood flow velocity as a blood flow index (BFI) based on the relationship between blood flow and light scattering. Studies have demonstrated the utility of DCS for measuring blood flow in organs, such as the muscles and brain, and its application in various conditions, including ischaemic brain disease and peripheral arterial disease [13, 14]. Skin and muscle blood flow may serve as early indicators of systemic microcirculatory dysfunction, as they are often sacrificed during acute circulatory failure [15]. Vishwanath et al. observed a decrease in BFI immediately below the skin in a haemorrhagic shock model [16]. However, the responsiveness of BFI to shock onset and its correlation with clinically used microcirculatory parameters remain unknown. We hypothesised that BFI could be useful in detecting microcirculatory dysfunction because it sensitively reflects changes in peripheral blood flow. Therefore, the relationship between BFI and microcirculatory parameters commonly used in clinical practice was investigated using a canine model of hypovolemic shock, in which microcirculation can be impaired.

## Methods

### Animals

The animals received humane care in compliance with the “Principles of Laboratory Animal Care” formulated by the National Society for Medical Research, and the “Guide for the Care and Use of Laboratory Animals” prepared by the Institute of Laboratory Animal Resources and published by the National Institutes of Health (NIH Publication No. 86–23, revised 1996). The protocol for this study was approved by the Institutional Animal Care and Use Committee at the National Cerebral and Cardiovascular Center, Suita, Japan (ID: 24,003).

This study examined six consecutive male Beagle dogs weighing 11.1 (10.9–11.4) kg [median (interquartile range)]. All dogs were housed in a room on a 12:12 h light/dark cycle with controlled temperature (22 ± 1 °C) and humidity (55 ± 15%). All dogs were considered healthy based on the medical history provided by the laboratory animal vendor and the findings of the physical examination performed by the investigators and the institutional veterinary care team. Prior to commencing the study, the dogs were familiarised with handling and laboratory conditions by animal caretakers and investigators. The dogs were fed commercial dog food and fresh water was available ad libitum. The experiment was conducted after a 14-day acclimatisation period. Food, but not water, was withheld for a minimum of 8 h before the induction of anaesthesia.

### Preparation

Anaesthesia was induced via the intravenous administration of propofol. All dogs were placed in the supine position after endotracheal intubation. Anaesthesia was maintained with 1.5% isoflurane in an oxygen–air mixture (flow rate: 2 L·min⁻^1^; fraction of inspired oxygen: 0.21–0.23; oxygen saturation of haemoglobin: 95–99%) using a semi-closed rebreathing system. The end-tidal carbon dioxide and oxygen saturation were monitored using a multiparameter biological information-monitoring device (Life Scope BSM-2391; Nihon Kohden Corp., Tokyo, Japan) with a built-in automatic calibration system. Mechanical ventilation was initiated immediately post-induction with a ventilator (A7, Shenzhen Mindray Bio-Medical Electronics Co., Ltd, Osaka, Japan) in A/C mode [volume-controlled, tidal volume: 10–15 mL·kg⁻^1^; respiratory rate: 16–25 breaths·min⁻^1^] to maintain eucapnia (end-tidal carbon dioxide: 35–45 mmHg). Rectal temperature was regulated at 37–38 °C using a forced-air patient warmer (Circulating Thermal Water System, T-CARE; Kimuramed, Tokyo, Japan).

**Fig. 1:**
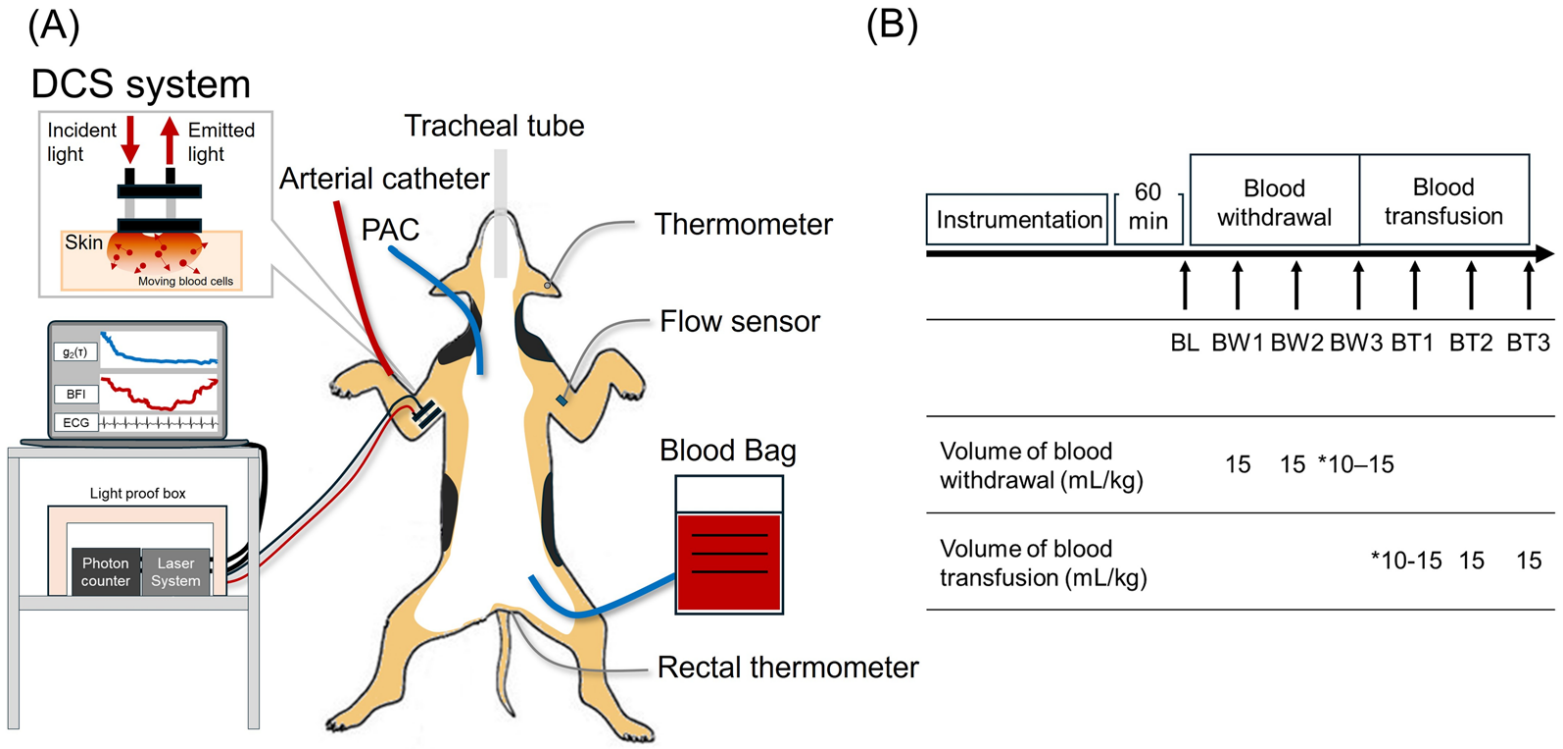
Overview of the experimental environment and protocol. **A** A catheter was placed in the right radial artery to measure MAP, while a 5 Fr pulmonary artery catheter was inserted through an 8 Fr sheath in the right external jugular vein. An incision was made on the proximal side of the left forelimb, and RAF was directly measured using a 1-mm flow sensor. The DCS system, equipped with a 785 nm continuous-wave laser and a photon counter, collected light intensity data at 1 MS/s, calculating BFI every second. The emitter and detector probes were positioned on the skin of the right forelimb. **B** Baseline measurements were obtained after a 1-h stabilisation period following splenectomy. Hypovolemic shock was induced by three sequential blood withdrawals (15 mL/kg over three minutes), followed by transfusion of the same volume. Measurements were taken after a 10-min stabilisation period following each withdrawal and transfusion. *If circulatory collapse occurred, as indicated by a significant decrease in HR, blood withdrawal was immediately halted, as necessary. DCS; diffuse correlation spectroscopy; BFI, blood flow index; PAC, pulmonary artery catheter; MAP, mean artery pressure; RAF, radial artery blood flow; BL, baseline; BW, blood withdraw; BT, blood transfusion

As shown in Fig. 1A, a 4 Fr sheath (BD Insyte-A; Becton, Dickinson & Co.) was inserted into the right radial artery and connected to a pressure transducer (DX-200; Nihon Kohden, Tokyo, Japan) via fluid-filled non-compliant tubing to measure and calculate the mean arterial pressure (MAP). A pulmonary artery catheter (5 Fr) was inserted through an 8 Fr sheath from the right external jugular vein with the tip positioned 1 cm from the wedge site near the pulmonary artery bifurcation. Lactated Ringer’s solution (Lactec; Otsuka Pharmaceutical Factory, Japan) was infused at 10 mL·kg⁻^1^·h⁻^1^ during the first hour, followed by 4 mL·kg⁻^1^·h⁻^1^ for the remainder of the study using an infusion pump (Terufusion TE-LM835A). A biological amplifier (AB-601G, AP-641G; Nihon Kohden) amplified the electrocardiogram (ECG), heart rate (HR), and arterial pressure signals, which were digitised at 200 Hz using a 16-bit analogue-to-digital converter (AD16-16 U (PCI) EV; Contec, Japan) and stored on a laboratory computer (LC-72 N10; Logitec, Tokyo, Japan) for offline analysis. Cefazolin (30 mL/kg) was administered to prevent surgical site infection. Given the dogs’ significant bleeding reserve from splenic contraction, splenectomy was performed via a small abdominal incision [17], followed by wound closure and a one-hour stabilisation period to ensure haemodynamic stability before baseline measurements. Pentazocine (15 mg) was administered for analgesia prior to cannulation and abdominal incision.

### Measurements

Surface ECG was recorded to monitor HR. The temperature of the auricle for skin temperature and the rectal temperature for central temperature were recorded, and the difference between the two was calculated as ΔT. MAP and central venous pressures (CVP) were measured throughout the experiment. Pulmonary artery wedge pressure (PAWP) was measured using balloon inflation and position adjustment as appropriate at a positive end-expiratory pressure of 5 mmHg at the end of the expiratory phase. The proximal side of the left forelimb was incised, and radial artery blood flow (RAF) was directly measured using a 1 mm-sized flow sensor. The pressure transducer was placed at the right atrium level as a reference point for zero pressure. All pressure and flow values were measured using a lab chart (v8.1.25) (LabChart 8; AD Instruments, Dunedin, New Zealand), and continuous measurements were averaged over five heartbeats.

In addition to the continuous monitoring of blood pressure and RAF, the following measurements were recorded intermittently on seven occasions: baseline after 1-h post-splenectomy stabilisation period, three 10-min blood withdrawal, and three 10-min blood returns. The shock index (SI), defined as heart rate divided by systolic blood pressure, at each measurement point was calculated. To measure CO using the thermodilution method, a bolus of 3 mL of cold water (0–4 °C) was administered three times, and the mean value was recorded. Haemoglobin concentration (Hb), serum lactate (lactate), and various blood oxygen saturations were assessed using arterial and pulmonary artery catheters. The veno-arterial difference in the partial pressure of carbon dioxide (PCO₂ gap) was calculated as the difference in carbon dioxide partial pressure between the mixed venous and arterial blood.

### Microvascular blood flow measurements

DCS measurements were obtained using a custom-built instrument, as previously reported [18, 19]. The DCS system emits near-infrared light onto the skin and detects scattered light from moving particles, such as red blood cells, within deep tissues. The intensity of the detected light was measured, and BFI was determined by calculating the autocorrelation function from the light intensity time course and fitting it to the theoretical autocorrelation function. As shown in Fig. 1A, the DCS probe was placed on the skin of the forelimb, where muscles were the predominant underlying tissue. Since the presence of bone or large vessels near the probe could affect measurement accuracy, the probe’s position was adjusted based on the initial values and the waveform of autocorrelation function before being fixed. This instrument was equipped with a 785 nm continuous-wave laser and a receiving probe connected to a photon counter. With a 2.5-cm distance between the source and detector fibres, the system facilitates light penetration through the skin to the muscle and skull (several centimetres). DCS collects light intensity data at 1 MS/s and determines BFI every second. Emitter and detector probes were positioned on the skin of the right forelimb. Since the haematocrit levels can affect optical constants, BFI was adjusted based on haematocrit values collected by blood gas analysis after each blood volume change from the baseline [20]. The BFI varies greatly with body size, sex, age, probe location, and species. Therefore, relative BFI (rBFI) was calculated as in previous studies, normalised by the mean of the baseline period [21].

### Protocol

Baseline measurements were obtained after a 1-h stabilisation period following splenectomy. Hypovolemic shock was induced by performing three sequential blood withdrawals (Fig. 1B) at a volume of 15 mL/kg over three minutes. Subsequently, the same volume of blood (15 mL/kg) was transfused sequentially over a period of three minutes. Shock was defined as a decrease in CO of at least 30% from baseline, with MAP < 60 mmHg or systolic arterial pressure (SAP) < 90 mmHg. After each blood withdrawal and transfusion, measurements were recorded after a 10-min stabilisation period. If circulatory collapse occurred, as indicated by a significant decrease in HR, blood withdrawal was immediately halted, as necessary. In these cases, the dogs were resuscitated using adrenaline. If 15 mL/kg of blood could not be absorbed during the third withdrawal owing to haemodynamic collapse, the same volume as the last withdrawal was transfused during the first transfusion.

### Statistical analysis

Statistical analyses were performed using R software (version 4.4.2, R Foundation for Statistical Computing, Vienna, Austria). Continuous variables are expressed as the median and interquartile range (IQR). The Friedman test was conducted using the stats package, and post hoc pairwise comparisons were performed with Conover’s test using the PMCMRplus package [22]. Holm’s method was used to adjust for type 1 errors owing to multiple comparisons. Correlations between BFI and ΔT, SvO₂, PCO₂ gap, lactate, RAF, and CO were analysed using the rmcorr package to account for repeated measurements, which estimates the common regression slope shared among individuals and calculated confidence intervals by a random intercepts model [23]. As the clearance of elevated lactate is dependent on its removal from the liver and kidneys, even after reperfusion [24], only data from baseline to the third blood withdrawal were used. Lactate and rBFI values from baseline to the third blood withdrawal were analysed using receiver operating characteristic (ROC) curves to determine the rBFI threshold for predicting an increase in the serum lactate levels above 22.5 mg/dL during bleeding which has been reported as the upper limit of the normal range in dogs [25]. As a sensitivity analysis, the same ROC analysis was performed in a subgroup of dogs, excluding those with serum lactate levels exceeding 22.5 mg/dL at baseline. The criterion for determining the optimal threshold on the ROC curve was defined as the threshold at which the sum of sensitivity and specificity is maximised. The statistical significance level was set at *p* < 0.05 (two-tailed test).

## Results

### Changes in individual haemodynamic and clinical microcirculatory parameters

**Fig. 2:**
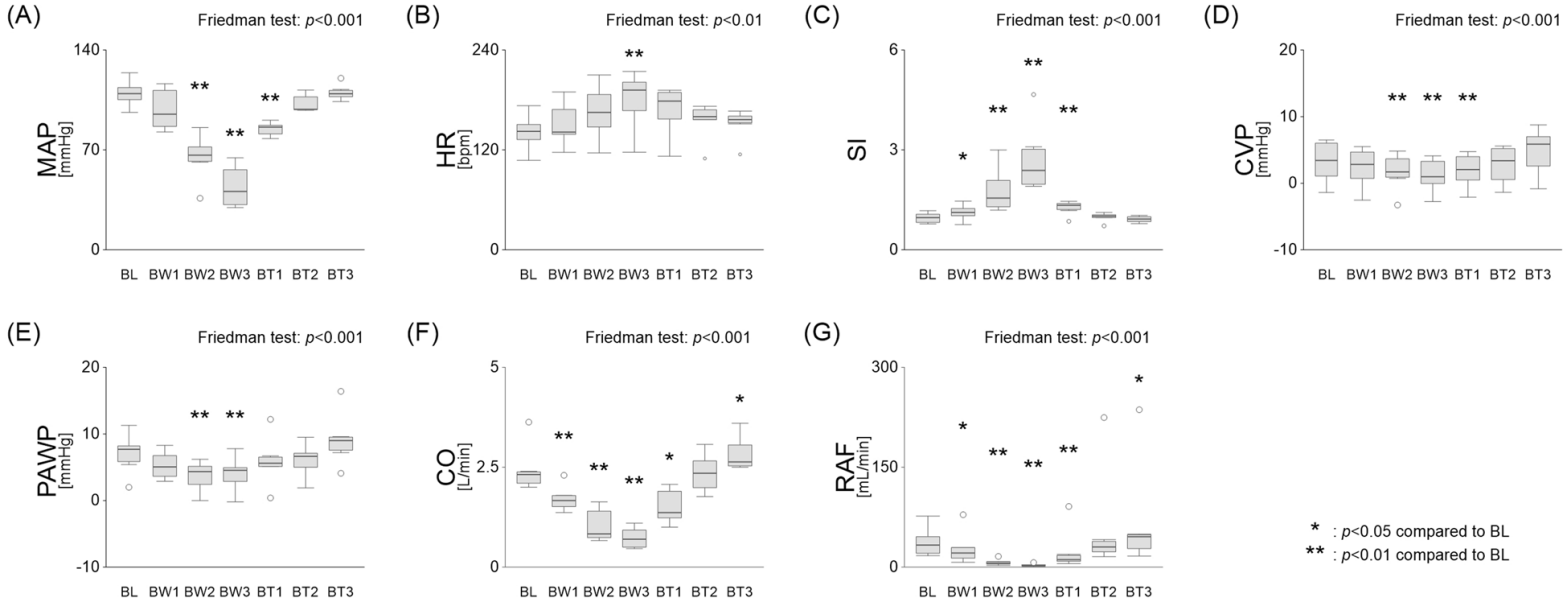
Changes in haemodynamic parameters during hypovolemic shock and resuscitation. **A**–**C** Three blood withdrawals significantly decreased MAP and increased HR and SI, indicating hypovolemic shock. **D**–**G** Blood withdrawal progressively reduced PAWP, CVP, CO, and RAF, all of which returned to baseline after transfusion. *p < 0.05, **p < 0.01 vs. baseline. MAP, mean artery pressure; HR, heart rate; SI, shock index; PAWP, pulmonary artery wedge pressure; CVP, central venous pressure; CO, cardiac output; RAF, radial artery blood flow; BL, baseline; BW, blood withdraw; BT, blood transfusion

Figure 2 shows changes in central and peripheral haemodynamic parameters during hypovolemic shock and resuscitation. Blood withdrawals significantly decreased MAP from baseline (BL) to the second and third withdrawals (BW), while blood transfusion (BT) improved MAP to almost baseline levels (BL: 109.5 [105.2–113.7], BW1: 95.1 [86.6–111.7], BW2: 66.3 [62.0–72.1], BW3: 40.9 [31.7–56.1], BT1: 86.0 [81.2–87.2], BT2: 98.4 [98.3–107.2], BT3: 109.4 [107.3–111.7] mmHg; Fig. 2A). HR progressively increased with each blood withdrawal, rising from 142.3 [132.5–150.6] at BL to a peak of 191.8 [167.2–201.4] at BW3. It then gradually declined to 156.4 [152.6–160.7] at BT3 (Fig. 2B). The SI increased progressively with each BW from 1.0 [0.8–1.1] at BL to a peak of 2.4 [2.0–3.0] at BW3, gradually declining to 0.9 [0.8–1.0] following the third transfusion (Fig. 2C). CVP dropped from 3.4 [1.1–6.0] mmHg at BL to 1.0 [0–3.3] mmHg at BW3, recovering to 5.9 [2.6–7.0] mmHg after the final transfusion (Fig. 2D). PAWP decreased from an initial 7.7 [5.8–8.2] mmHg to 4.5 [2.8–4.9] mmHg at BW3, then rebounded to 9.0 [7.5–9.5] mmHg post-transfusion (Fig. 2E). CO decreased from 2.3 [2.1–2.4] L/min initially to 0.7 [0.5–0.9] L/min at BW3, before rising to 2.6 [2.5–3.1] L/min post-transfusion (Fig. 2F). Similarly, RAF diminished from 33.2 [20.9–45.8] mL/min at BL to a low of 2.5 [2.4–3.3] mL/min at BW3, with a notable recovery to 45.7 [27.9–48.7] mL/min after the third transfusion (Fig. 2G).

**Fig. 3:**
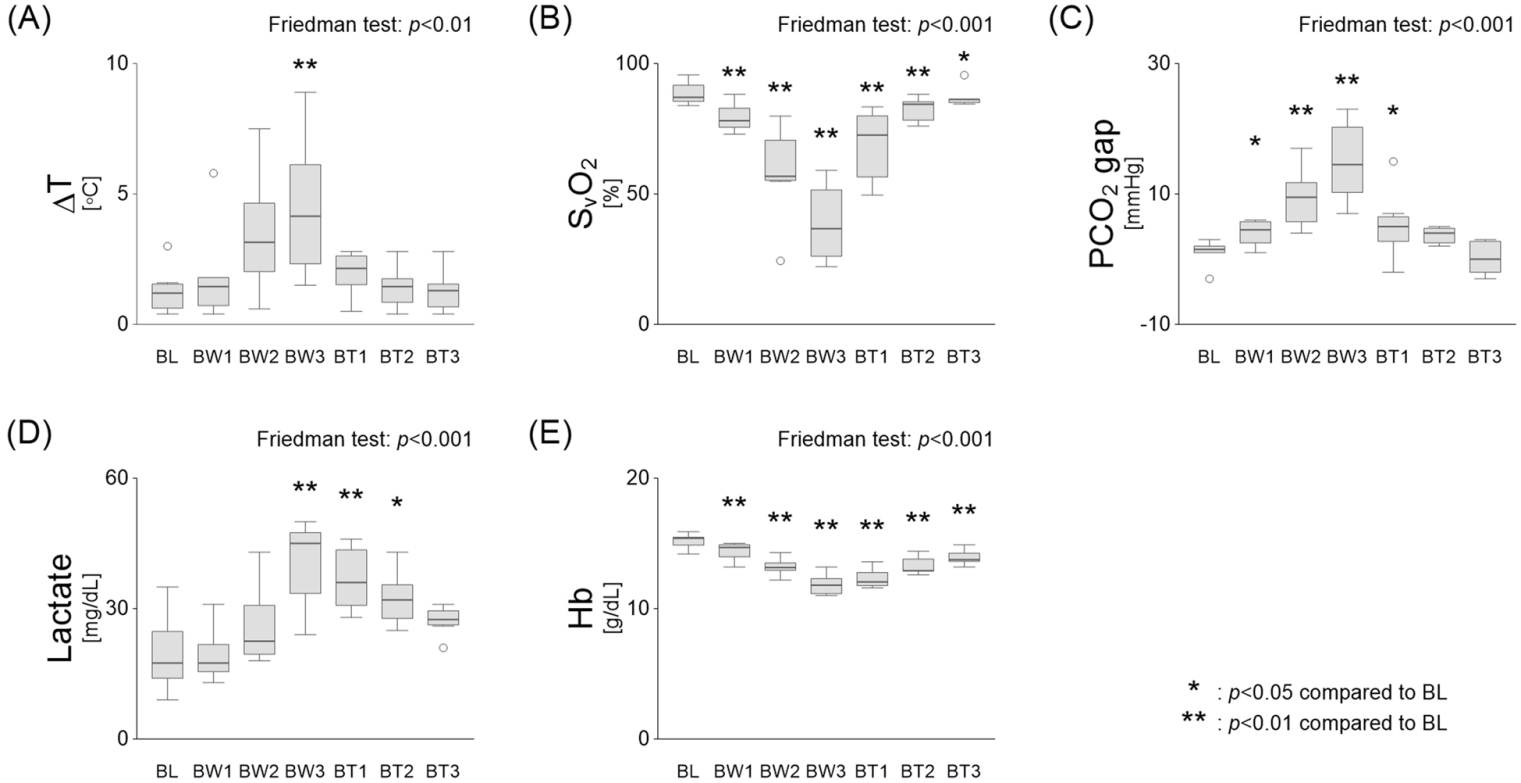
Changes in microcirculatory parameters during hypovolemic shock and resuscitation. **A**–**C** Clinical microcirculatory parameters, including ΔT, S_v_O_2_, PCO_2_ gap, deteriorated with blood withdrawal and showed recovery after transfusion. **D** Serum lactate levels decreased during blood withdrawal but increased following transfusion, remaining higher than baseline. **E** Haemoglobin levels dropped during blood withdrawal and nearly returned to normal after transfusion. *p < 0.05, **p < 0.01 vs. baseline. ΔT, core-to-skin temperature gradient; S_v_O₂, mixed venous oxygen saturation; PCO_2_ gap, veno-arterial difference in partial pressure of carbon dioxide; Hb, haemoglobin concentration; BL, baseline; BW, blood withdrawal; BT, blood transfusion

Clinical microcirculatory parameters, including ΔT, S_v_O₂, PCO_2_ gap, and serum lactate levels, deteriorated with blood withdrawal and recovered after transfusion (Fig. 3). However, the serum lactate levels improved post-transfusion but remained elevated compared to baseline levels. The haemoglobin levels decreased during blood withdrawal, but nearly normalised post-transfusion. During blood withdrawal, two dogs showed haemodynamic compromise after the BW2 and could not undergo the third challenge, resulting in a blood volume loss below 15 mL/kg. For these dogs, the first transfusion volume was adjusted to the third withdrawal volume (dog #5:10 mL/kg; dog #6:12 mL/kg), whereas the second and third transfusions were adjusted to 15 mL/kg each.

### Changes in BFI and rBFI

**Fig. 4:**
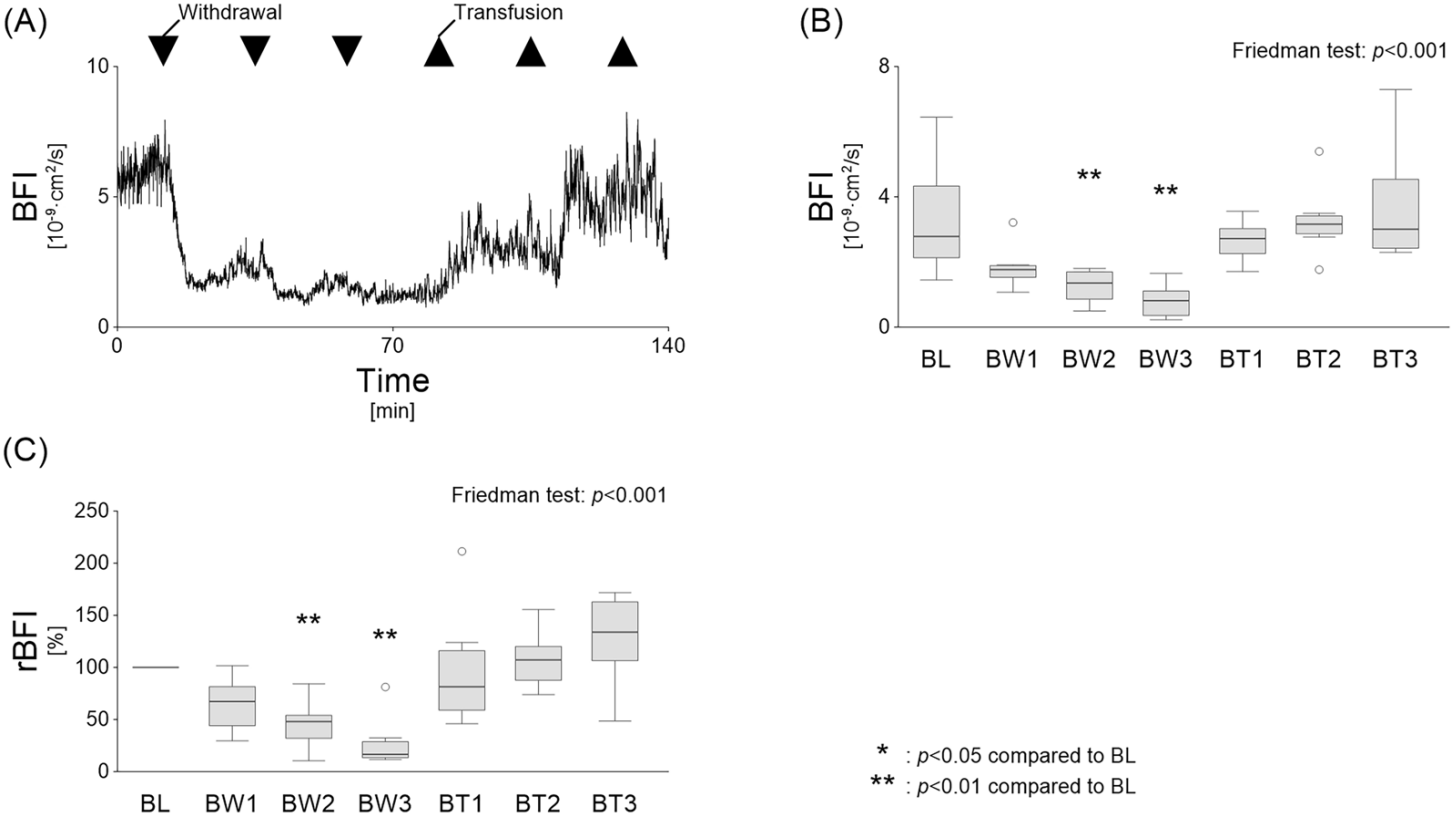
Changes in BFI and rBFI during hypovolemic shock and resuscitation. **A** Representative time course of BFI during blood withdrawal and transfusion demonstrates an immediate decrease with blood withdrawal and a recovery with transfusion. **B** The box plots show that BFI decreased during blood withdrawal, with values after BW2 and BW3 significantly lower than baseline. Transfusion restored BFI to near-baseline levels. **C** Similarly, rBFI, which represents BFI relative to baseline, also showed a significant decline during blood withdrawal and recovered to baseline levels after transfusion. **p < 0.01 vs. baseline. BFI, blood flow index; rBFI, relative blood flow index; BL, baseline; BW, blood withdrawal; BT, blood transfusion

As shown in the representative time course, BFI decreased immediately after blood withdrawal and increased with transfusion (Fig. 4A). The box plots show that BFI decreased during blood withdrawal, with values after BW2 and BW3 significantly lower than baseline (BL: 2.8 [2.1–4.3], BW2: 1.4 [0.9–1.7], BW3: 0.8 [0.4–1.1]; each *p* < 0.01). Transfusion restored BFI to near-baseline levels (BT3: 3.0 [2.4–4.5]) (Fig. 4B). Similarly, rBFI, which represents BFI relative to baseline, also showed a significant decline during blood withdrawal (BW2: 48.1 [32.1–54.0], BW3: 16.7 [13.4–28.8]; each *p* < 0.01) and recovered to baseline levels after transfusion (Fig. 4C).

### Correlation of BFI with blood flows and clinical microcirculatory parameters

A correlation analysis accounting for repeated measurements revealed significant associations between BFI and both CO (correlation coefficient [CC] = 0.82, 95% confidence interval [CI 0.68 to 0.91, *p* < 0.01) and RAF (CC = 0.41, 95% CI 0.10 to 0.65, *p* = 0.01).

**Fig. 5:**
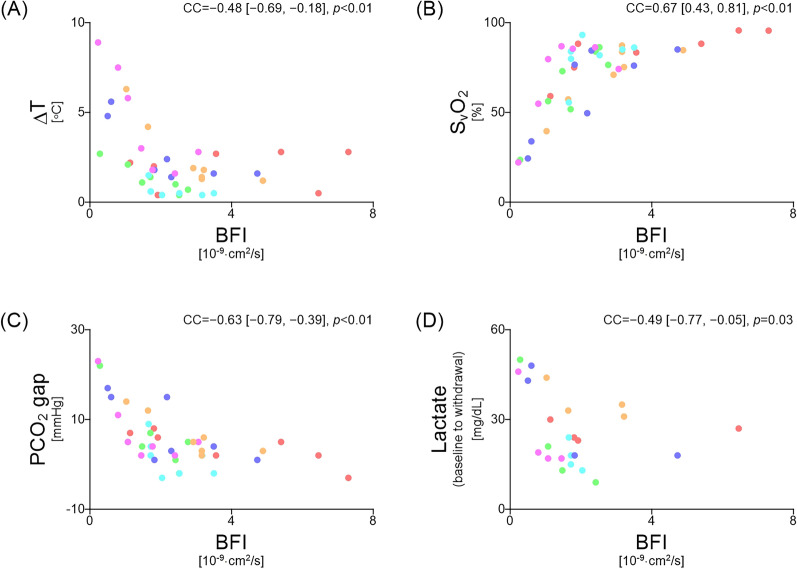
Correlation analysis, accounting for repeated measurements, between BFI and clinical microcirculatory parameters. **A**–**C** BFI correlated significantly with clinical microcirculatory parameters, with ΔT, S_v_O_2_ and PCO_2_ gaps showing moderate correlations. **D** Serum lactate levels also showed a significant correlation with the BFI from baseline to the third withdrawal. Values enclosed in quotation marks indicate the 95% confidence interval. CC, correlation coefficient; BFI, blood flow index; ΔT, core-to-skin temperature gradient; S_v_O_2_, mixed venous oxygen saturation; PCO_2_ gap, veno-arterial difference in partial pressure of carbon dioxide

Figure 5 shows the correlations, accounting for repeated measurements, between BFI and clinical microcirculatory parameters at BL and after three blood withdrawals and transfusions. Significant correlations were observed between changes in BFI and ΔT (CC = − 0.48, 95% CI − 0.69 to − 0.18, *p* < 0.01), SvO₂ (CC = 0.67, 95% CI 0.43 to 0.81, *p* < 0.01), the PCO₂ gap (CC = − 0.63, 95% CI − 0.79 to − 0.39, *p* < 0.01), and serum lactate (CC = − 0.49, 95% CI − 0.77 to − 0.05, *p* = 0.03).

**Fig. 6:**
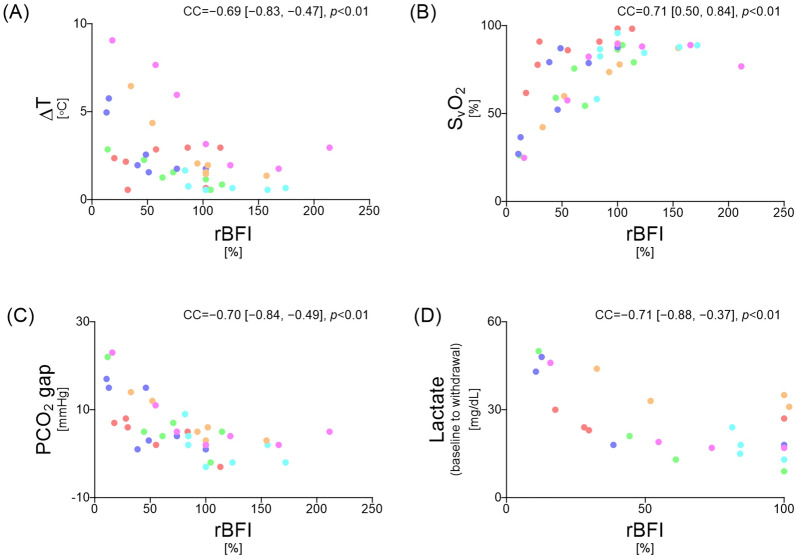
Correlation analysis, accounting for repeated measurements, between rBFI and clinical microcirculatory parameters. **A**–**C** rBFI correlated significantly with clinical microcirculatory parameters, with ΔT, S_v_O_2_, and PCO_2_ gaps showing moderate correlations. **D** Serum lactate levels also showed a significant correlation with the rBFI from baseline to the third withdrawal. Values enclosed in quotation marks indicate the 95% confidence interval. CC, correlation coefficient; rBFI, relative blood flow index; ΔT, core-to-skin temperature gradient; S_v_O_2_, mixed venous oxygen saturation; PCO_2_ gap, veno-arterial difference in partial pressure of carbon dioxide

**Fig. 7:**
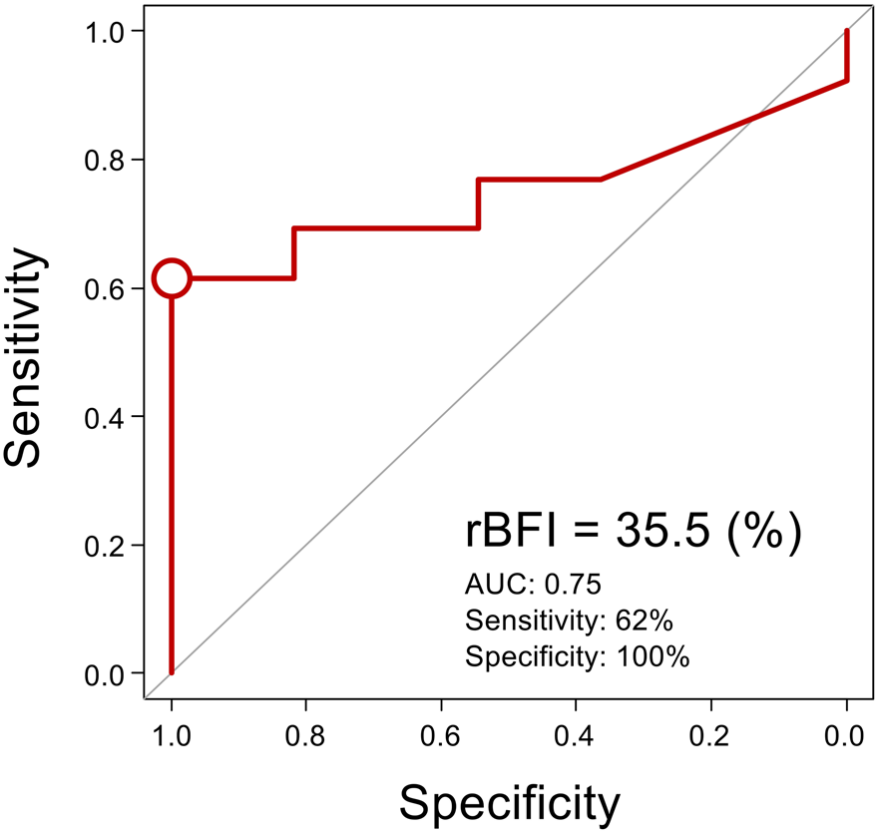
ROC curve analysis to determine the rBFI cut-off for predicting abnormal lactate levels. ROC curve analysis identified an rBFI threshold of 35.5% of the reference value for predicting increased serum lactate during bleeding, with a sensitivity of 62%, specificity of 100%, and an AUC of 0.75. ROC, receiver operating characteristic; rBFI, relative blood flow index; AUC, area under the curve

**Fig. 8:**
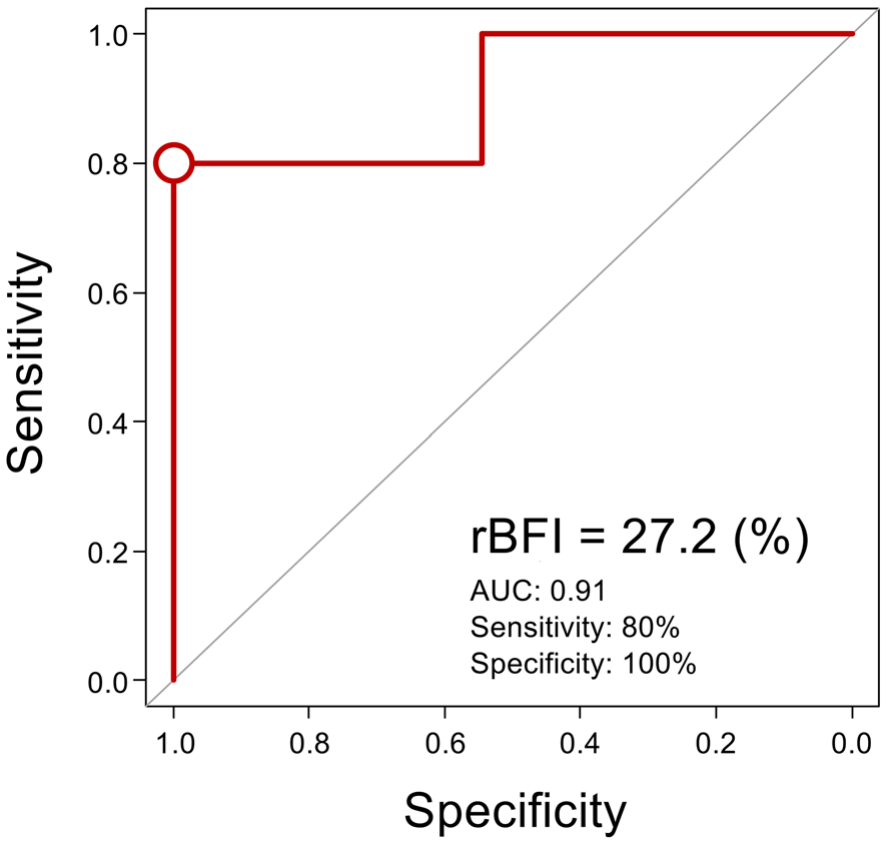
ROC curve analysis to determine the rBFI cut-off for predicting abnormal lactate levels, excluding two dogs. ROC curve analysis identified an rBFI threshold of 27.2% of the reference value for predicting increased serum lactate during bleeding, with a sensitivity of 80%, specificity of 100%, and an AUC of 0.91. ROC, receiver operating characteristic; rBFI, relative blood flow index; AUC, area under the curve

Figure 6 presents the correlations, also considering repeated measurements, between rBFI and clinical microcirculatory parameters at the same time points as in Fig. 5. Significant correlations were observed between rBFI and all microcirculatory parameters: ΔT (CC = − 0.69, 95% CI − 0.83 to − 0.47, *p* < 0.01), SvO₂ (CC = 0.71, 95% CI 0.50 to 0.84, *p* < 0.01), PCO₂ gap (CC = − 0.70, 95% CI − 0.84 to − 0.49, *p* < 0.01), and serum lactate (CC = − 0.71, 95% CI − 0.88 to − 0.37, *p* < 0.01). As shown in Fig. 7, ROC curve analysis identified an rBFI threshold of 35.5% of the reference value for predicting increased serum lactate (≥ 22.5 mg/dL) during blood withdrawal, with a sensitivity of 62%, specificity of 100%, and an AUC of 0.75. The two dogs already had elevated baseline serum lactate levels (dog #1: 27 mg/dL, dog #2: 35 mg/dL). A sensitivity analysis excluding these two cases was performed on the remaining four cases, resulting in an rBFI threshold of 27.2%, a sensitivity of 80%, a specificity of 100%, and an AUC of 0.91 (Fig. 8).

## Discussion

This study investigated changes in microcirculatory haemodynamics during hypovolemic shock using DCS, which quantifies deep tissue blood flow as a BFI. The main findings were as follows: (1) changes in BFI resulting from blood withdrawal and transfusion showed a significant correlation with several clinical microcirculatory parameters; (2) dynamic changes in peripheral blood flow during blood withdrawal and transfusion were reflected in the BFI.

### Relationship between BFI and clinical microcirculatory parameters

In hypovolemic shock, BFI changed from baseline after blood withdrawals and transfusions and showed a moderate correlation with widely used microcirculatory parameters, supporting its potential as a novel and reliable microcirculatory parameter (Fig. 5). A previous study indicated that the lactate levels were found to gradually increase as oxygen delivery dropped to approximately half of the normal levels [26]. In this study, the rBFI threshold for predicting abnormal lactate (> 22.5 mg/dL) was relatively low at 35.5% (Fig. 7). The two dogs already had elevated baseline serum lactate levels possibly due to their pre-experimental dehydration status. Therefore, as a sensitivity analysis, a similar ROC analysis excluding these two cases was performed, resulting in an rBFI threshold of 27.2%, a sensitivity of 80%, and a specificity of 100% (Fig. 8). The significantly low rBFI value for predicting abnormal lactate suggests that skin and muscle blood flow is highly vulnerable to sacrifice during hypovolemic shock.

Insufficient oxygen delivery to tissues causes an oxygen supply–demand mismatch, triggering anaerobic metabolism, and leading to organ dysfunction [27]. In recent years, the focus has shifted towards a tissue-centric approach as a key strategy for managing circulatory shock, moving away from the traditional emphasis on increasing global oxygen delivery [28]. Tissue oxygenation is primarily influenced by microvascular blood flow, haemoglobin concentration, and oxygen saturation [29]. In most patients with shock, arterial oxygen saturation is maintained within the normal range, with the exception of cases with severe cardiogenic pulmonary oedema or pneumonia, making tissue blood flow a critical factor in differentiating tissue oxygenation failure.

Mixed venous oxygen saturation reflects tissue oxygen utilisation but may not indicate microcirculatory dysfunction in specific diseases, such as septic shock [30]. Elevated lactate levels can also result from insufficient perfusion, liver dysfunction, toxins, drugs, or metabolic factors. In addition, the existence of temporal time lags between acute changes in tissue perfusion and lactate levels are well documented [31]. In our study, rBFI decreased at BW1 (Fig. 4), prior to the increase in lactate observed at BW2 (Fig. 3). This suggests that rBFI may serve as a more responsive indicator of microcirculatory deterioration compared to lactate. Although laser Doppler flowmetry is useful for continuously assessing peripheral blood flow, it has limitations, including poor reproducibility owing to small tissue volumes and sensitivity to motion artefacts and environmental factors [32]. On the other hand, DCS may be more tolerant to environmental changes compared to skin blood flow measurements, as it reflects blood flow changes in deep tissues, reaching several centimetres below the skin. Compared to current microcirculatory parameters, which indirectly reflect tissue oxygen metabolism, DCS plays a strong role in directly assessing microvascular blood flow. To the best of our knowledge, no previous studies have investigated the relationship between BFI and clinical microcirculatory parameters, making this study the first to demonstrate that DCS can be used to assess microcirculatory deterioration due to peripheral perfusion decline caused by haemorrhage.

While all dogs received the same infusion protocols, their responses to blood withdrawal varied significantly, likely due to differences in pre-experiment dehydration status and individual vascular reactivity. Among the factors to consider, the stepwise blood withdrawal in this study may have influenced the results by causing a time-dependent decrease in tissue perfusion. Therefore, establishing an appropriate rBFI threshold for predicting abnormal lactate levels may require alternative protocols, such as monitoring after a single blood withdrawal or multiple small-volume withdrawals. Additionally, accumulating clinical data on BFI and lactate levels will be crucial for determining the optimal threshold.

### Impact of hypovolemic shock on BFI

Blood withdrawal caused rapid and significant decreases in BFI and RAF, which were restored by transfusions (Figs. 2 and 4). This indicates that BFI is a sensitive marker of peripheral blood flow changes, offering continuous quantitative microcirculatory assessments. Previous studies have highlighted the utility of DCS for monitoring peripheral blood flow. Nakabayashi et al. reported that decreased blood flow with tourniquet occlusion to the arm reduced BFI, which returned to baseline upon release [18]. Similarly, Vishwanath et al. demonstrated that DCS effectively detects vascular responses in a porcine haemorrhagic shock model [16]. Hypovolemic shock decreases blood pressure and activates the sympathetic nervous system through baroreceptor reflexes, leading to microvascular constriction in skin and muscles [33]. While rapid assessment of these microcirculatory changes is crucial, reliable clinical parameters have yet to be established. Many existing parameters indirectly reflect oxygen metabolism and lack the sensitivity and continuity required to detect microcirculatory dysfunction. In this context, the continuous visualisation of peripheral blood flow using DCS is a valuable tool for the rapid detection of tissue perfusion deficits and may contribute to the optimisation of microcirculatory management in critically ill patients.

Although DCS appears to detect changes in tissue blood flow immediately in the representative case (Fig. 4A), statistical analysis revealed a significant decrease in BFI compared to baseline only after the second blood withdrawal (Fig. 4B and C). This delay may be attributed to individual variations in baseline microcirculatory conditions, vascular responses, and the small sample size. In addition, despite receiving three transfusions and achieving restored macrocirculatory parameters, one dog exhibited persistently low rBFI at 49%. Previous studies suggest that microcirculatory impairment can persist even after macrocirculatory restoration, possibly due to mitochondrial dysfunction [7, 34]. Thus, DCS may help clinicians assess treatment responsiveness by visualising changes in peripheral blood flow. Further research is needed to assess BFI's responsiveness over time in detecting microcirculatory changes.

### Needs for device development using DCS technologies

Microcirculatory parameters commonly used in intensive care units include serum lactate levels, PCO_2_ gap, core-to-skin temperature gradient, and near-infrared spectroscopy (NIRS). While these indicators primarily evaluate tissue oxygen metabolism and the adequacy of peripheral perfusion, few directly measure peripheral blood flow. A novel device able to directly and continuously monitor peripheral perfusion has long been sought to facilitate the early diagnosis of microcirculatory dysfunction. Previous studies have demonstrated the value of DCS in evaluating cerebral perfusion [13, 35]. The application of DCS to both the head and arms may enable the simultaneous assessment of macro- and microcirculation. Furthermore, advances combining DCS with NIRS (DCS-NIRS) show promise for assessing tissue oxygen consumption, potentially improving tissue oxygenation management [36, 37]. The experimental strength of DCS is its ability to measure tissue blood flow under non-pulsatile haemodynamic conditions [19]. This unique feature is particularly beneficial in severe cardiogenic shock, where low cardiac output and the use of non-pulsatile mechanical circulatory support, such as VA-ECMO or percutaneous left ventricular assist devices, make the quantitative assessment of peripheral perfusion challenging. Theoretically, only a few devices, such as DCS and laser Doppler flowmetry, may be able to continuously measure peripheral blood flow in such situations. However, the role of DCS in shock states beyond hypovolemic shock remains unknown. Future research should investigate the clinical significance of peripheral blood flow assessment using non-invasive devices, including DCS and laser Doppler flowmetry, in severe cardiogenic shock.

### Limitations

This study has several limitations. First, the sample size was small. Additionally, given significant bleeding reserve from splenic contraction in dogs [17], splenectomy was performed before blood withdrawal in this study. However, splenectomy itself may have triggered an acute systemic inflammatory response, with leukocyte-derived mediators such as histamine and bradykinin potentially affecting microcirculation by inducing arteriolar dilation and increasing peripheral blood flow [38]. While all dogs received the same infusion protocols, their responses to blood withdrawal varied significantly, likely due to differences in pre-experiment dehydration status and individual vascular reactivity. Furthermore, although the blood withdrawal protocol included active warming to maintain rectal temperature, clinical patients often present with hypothermia. Systemic warming may also impact microcirculation, as warming during haemorrhage could reduce both systemic blood flow and tissue perfusion [39]. Although there are no previous foundational studies on DCS and microcirculatory dysfunction, the results of this study suggest that DCS is feasible for detecting microcirculatory dysfunction. Second, the calculation of BFI involves approximations, and in cases where the diffusion equation for a semi-infinite line is not valid or when the tissue is non-uniform, the BFI values may vary. Additionally, the BFI values were found to be influenced by factors such as species, measurement site, and fluid status of the body. As previous studies have suggested, rBFI, which represents the relative change compared with the baseline BFI, proved useful in comparison with microcirculatory parameters. Currently, normal BFI values in humans remain unknown; however, future clinical studies incorporating these values are expected to improve the accuracy of detecting microcirculatory changes. Third, while DCS showed trends similar to those of RAF (Fig. 7) and correlated significantly with microcirculatory parameters in this study, we did not directly observe peripheral blood flow changes. Previous studies using direct monitoring devices, such as handheld vital microscopy, have shown that the microcirculation varies across different shock states and disease conditions [10]. In the future, research should focus on clarifying how DCS can be used to detect various types of microcirculatory dysfunctions.

## Conclusion

Blood flow index measured by DCS reflected peripheral perfusion changes due to blood withdrawal and transfusion. Moreover, it was significantly correlated with clinical microcirculatory parameters. Taken together, the results of this study demonstrate that non-invasive, peripheral perfusion assessment using DCS may be useful for detecting microcirculatory dysfunction in a hypovolemic shock model. Further investigation is required to clarify the time responsiveness of DCS in detecting microcirculatory changes.

## Data Availability

All relevant data obtained and analysed in this study are presented in the main text.

## Abbreviations

AUC: Area under the curve
BFI: Blood flow index
BL: Baseline
BP: Blood pressure
BW: Blood withdrawal
BT: Blood transfusion
CO: Cardiac output
CC: Correlation coefficient
CVP: Central venous pressures
DCS: Diffuse correlation spectroscopy
ΔT: Core-to-skin temperature gradient
ECG: Electrocardiogram
Hb: Haemoglobin concentration
HR: Heart rate
MAP: Mean arterial pressure
NIRS: Near-infrared spectroscopy
PCO_2_ gap: Veno-arterial difference in partial pressure of carbon dioxide
RAF: Radial artery blood flow
rBFI: Relative blood flow index
ROC: Receiver operating characteristic
SD: Standard deviation
SI: Shock index
S_v_O_2_: Mixed venous oxygen saturation
